# Stochastic modelling of the effects of human-mobility restriction and viral infection characteristics on the spread of COVID-19

**DOI:** 10.1038/s41598-021-86027-2

**Published:** 2021-03-25

**Authors:** Shiho Ando, Yuki Matsuzawa, Hiromichi Tsurui, Tetsuya Mizutani, Damien Hall, Yutaka Kuroda

**Affiliations:** 1 Department of Biotechnology and Life Sciences, Tokyo University of Agriculture and Technology, 2-24-16 Nakamachi, Koganei-shi, Tokyo, 184-8588 Japan; 2 Department of Immunological Diagnosis, Juntendo University School of Medicine, 2-1-1 Hongo, Bunkyo-ku, Tokyo, 113-8421 Japan; 3 Research and Education Center for Prevention of Global Infectious Diseases of Animals, Tokyo University of Agriculture and Technology, 3-5-8 Saiwai-Cho, Fuchu-shi, Tokyo, 183-8509 Japan; 4 Department of Life Sciences and Applied Chemistry, Nagoya Institute of Technology, Gokiso Showa, Nagoya, Aichi 466-8555 Japan; 5 WPI Nano Life Science Institute, Kanazawa University, Kakumamachi, Kanazawa, Ishikawa 920-1164 Japan; 6 Department of Applied Physics, Alto University, FI-00076 Aalto, Finland

**Keywords:** Computational biology and bioinformatics, Computational models

## Abstract

After several months of "lockdown" as the sole answer to the COVID-19 pandemic, balancing the re-opening of society against the implementation of non-pharmaceutical measures needed for minimizing interpersonal contacts has become important. Here, we present a stochastic model that examines this problem. In our model, people are allowed to move between discrete positions on a one-dimensional grid with viral infection possible when two people are collocated at the same site. Our model features three sets of adjustable parameters, which characterize (i) viral transmission, (ii) viral detection, and (iii) degree of personal mobility, and as such, it is able to provide a qualitative assessment of the potential for second-wave infection outbreaks based on the timing, extent, and pattern of the lockdown relaxation strategies. Our results suggest that a full lockdown will yield the lowest number of infections (as anticipated) but we also found that when personal mobility exceeded a critical level, infections increased, quickly reaching a plateau that depended solely on the population density. Confinement was not effective if not accompanied by a detection/quarantine capacity surpassing 40% of the symptomatic patients. Finally, taking action to ensure a viral transmission probability of less than 0.4, which, in real life, may mean actions such as social distancing or mask-wearing, could be as effective as a soft lockdown.

## Introduction

COVID-19 disease is an ongoing pandemic that was initially identified in Wuhan, China, in December 2019^1,2^. Until the time of writing (July 2020)), over fourteen million (updated to 86 millions on January 2021**)** COVID-19 patients have been reported worldwide, resulting in more than 600,000 (Updated to 1.86 million) fatalities^3^. COVID-19 infection is thought to occur from close person-to-person contact^4^. This has led most countries to enforce some kind of confinement policy to reduce interpersonal contact, thereby slowing the spread of infection in the absence of a specific medical anti-COVID-19 treatment^5^.

The causative agent of COVID-19 is *SARS-Cov2 (Severe Acute Respiratory Syndrome Coronavirus 2),* a lethal member of the *Coronaviridae* family which has been a focus of much attention since 2002^6,7^. *SARS-CoV2* is thought to be transmitted through encounters of small droplets produced by coughing or sneezing by an infected patient and dispersed either in the air (aerosols) or on surfaces (fomites)^5^. Aerosols are either directly breathed in or lodged within the eye, whilst in the case of fomites, the virus infects through touching the face with contaminated hands^5^. Common COVID-19 symptoms include cough, fever, shortness of breath, and loss of smell^1^. In a limited number of cases, serious complications can develop, including pneumonia and severe acute respiratory syndrome (*SARS*)^8^, leading to a high mortality rate. Typically, symptoms appear around five days after the time of infection. However, it can take longer, and up to 80% of the patients may show no, or only mild, symptoms^8^. Although COVID-19 patients are most infectious within the first few days following the onset of symptoms (post-symptomatic), both presymptomatic and asymptomatic patients are infectious to some extent^9^. As no anti-COVID-19 treatment is currently available, non-pharmaceutical interventions based on isolation of both infected and non-infected people have, far and away, been the primary means of avoiding the spread of the virus^4,10^.

After several months of confinement, many countries are progressively restarting their economic and social activities. To avoid further spreading of the virus, the re-opening of the economy should ideally proceed in a fashion that minimizes interpersonal contacts. Thus, it is necessary to assess the risks of infection associated with different relaxation strategies^10–12^. This paper introduces a stochastic model for the relaxation of lockdown that considers the re-opening of society in terms of people moving between sites on a one-dimensional grid. Our model contains three adjustable parameters characterizing viral transmission, viral detection, and degree of personal mobility. The model predicts that a full lockdown yields the best results (in line with general expectations) i.e., the lowest number of total infections. A less anticipated result was that when personal mobility is increased beyond a critical level, the risk of infection rapidly reaches a constant value, which depends solely on the population density.

Furthermore, according to our model, confinement alone is not effective if it is not accompanied by a detection capacity (coupled with quarantine) that surpasses 40% of the patients during their symptomatic phase. Our simulation results also showed that keeping the virus transmission probability to less than 0.4, which can be achieved in real life by respecting social distancing or wearing masks, is as effective as imposing a soft lockdown. Finally, we note that detection and quarantine of presymptomatic patients, even with a probability as low as 0.2, would reduce the final number of infections by a factor of ten or more. The results of the model should help in the qualitative assessment and semi-quantitative ranking of the importance of factors to be considered in the process of ‘restarting’ social and economic activities.

## Methods

**Figure 1:**
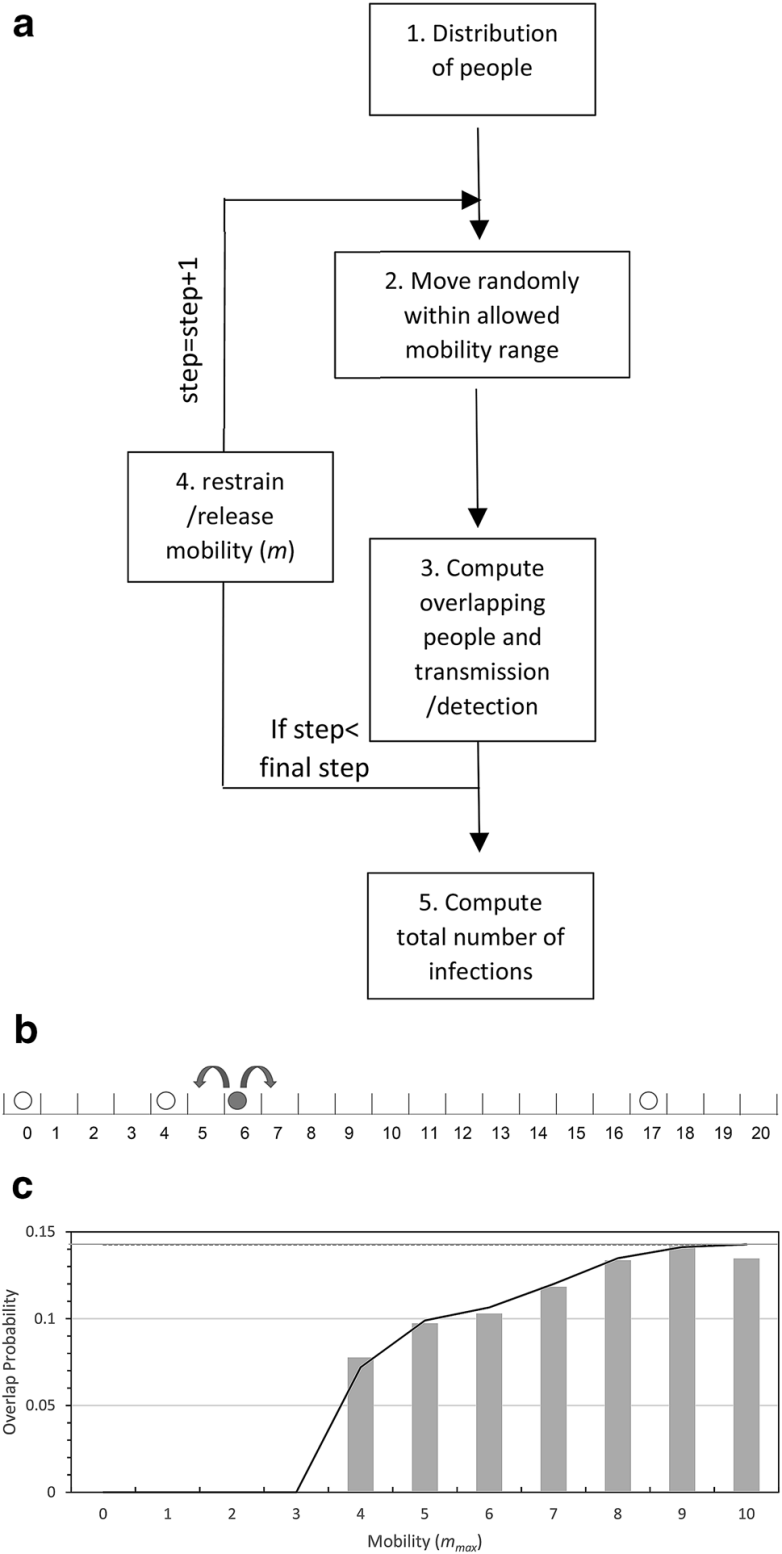
(**a**) Schematic algorithm of the simulation. The main steps are as follows: 1. *M* (= 1000) people are distributed on a one -dimensional grid having *N* sites (= 2000). 2. They can move freely within a window of + /− *m* sites (*m* was set to 100 when no confinement was enforced). *m* = 0 means that people were not allowed to move from their sites. 3. The people collocated at the same site were identified. People collocated with an infectious person were infected with a probability of *T.P.*, and the infected people were detected with a set detection probability (*D.P.*) and isolated from the system (detected people cannot infect nor be infected). 4. When the detected number of infected people reached 1% of the total number of all people (1% of 1000 people in our setting), *m* was decreased to the values indicated in Table 1. Infected people remained infectious for a total of 15 steps: 5 steps as a presymptomatic patient, and then ten steps as a symptomatic patient. Furthermore, we assumed that the virus would disappear after 15 steps, and that the infection occurs only once. (**b**) Schematics of the one-dimensional grid model with four people and 21 sites. The black and white circles represent the infected and the non-infected person, respectively. The arrows illustrate the possible movement of the infected person. For *m* = 1, the person can move by − 1, 0, or + 1. Viral transmission occurs with a probability of *T.P.* when an infected person occupies the same sites as a non-infected person. The periodic boundary condition implies that a person at position 21 can move to its right and will "reappear" at position 0 (and vice versa). It is used to alleviate the effects of the boundaries. (**c**) Comparison of the contact probability calculated using an exact probabilistic model (supplemental), and numerically from our simulation (average over 3000 runs with *N* = 3, *M* = 21). In the initial distribution, people were regularly-spaced at positions 0, 7, and 14 on the one-dimensional grid with a periodic boundary condition (between positions 0 and 20). People were then allowed to move according to any site elements within the mobility limit set to *m*_max_. The blue bars represent the results of the simulation, the red line those of the exact probabilistic mode, and the gray line represents the average probability of encounter for a random distribution (0.1428 for *N* = 3 people on a grid with *M* = 21 sites.

To simulate a population under lockdown, we used a one-dimensional grid containing *M* sites into which *N* ‘people’ were distributed in either a regular periodic fashion or randomly without exclusion (Fig. 1). During the lockdown period, each person’s mobility was restricted, and they were only allowed to move by a random distance (*m*) equal to or less than the maximum range of mobility (*m*_max_). Thus, *m*_max_ = 0 represents a full lockdown, whereas *m*_max_ = 1 describes the case where people can, with equal likelihood, either stay on the same site or move by one site to either the left or to the right. Un-restricted mobility is attained when *m*_max_ ≥ (*M* − 1)/2, (an odd number is used for *M* to avoid non-integer movements). Each movement was considered to take place in a time period *Δt*. The total time from the beginning of the lockdown was thus *t* = *Δt j*, where *j* is the number of periods completed up to time, *t*. A periodic boundary condition was applied to the running value of each *i*^th^ person’s position coordinate to suppress effects related to the finite size of the grid (Figure S1). Namely, (*x*_*i*_)_periodic_ = (*M* – |*x*_*i*_|) for *x*_*i*_ < 0; and (*x*_*i*_)_periodic_ =|*x*_*i*_ − *M*|for *x*_*i*_ > *M*. Simulations were run for *S* steps, and the results were averaged over *I* iterations. The following parameters were used to characterize viral transmission during the relaxation of lockdown.

(i) **Transmission Probability (*T.P.*):** *T.P.* describes the fractional probability of infection of a previously non-infected person by an infected person when the two are co-located simultaneously at the same grid site over a unit time, *Δt*.
(ii) **Infection Period (*I.P.*):** An infected person was considered to remain infectious for a given number of time intervals denoted by ***I.P.***, after which the person was no longer considered infectious and considered to have acquired immunity (i.e., they can't be infected a second time). Note that this study does not specify whether the patient recovers or not during or at the end of the *I.P.*, as it is simply set apart from the system (footnote 1^13–15^).
(iii) **Presymptomatic Phase (*P.P.*):** We assumed that following infection, *SARS-COV2* in infected people could not be detected (or detected with a lower sensitivity) for a specific period of time, termed the presymptomatic phase (*P.P.*). In this report, we set *P.P.* to a period of 5*Δt* after the initial infection. Our model considers, in line with current medical observations, that infected patients can transmit the virus during this presymptomatic phase^10^.
(iv) **Symptomatic Phase (*S.P.*):** The period of the infection cycle from when the patient started to exhibit symptoms up until the illness was resolved, was termed the symptomatic phase. The *S.P.* period was considered to last for 10*Δt*, beginning after *P.P.* period of 5*Δt* and lasting until the end of the infection period (*I.P.*), so that the total period of infection (*I.P.*) is 15*Δt.*
(v) **Detection Probability (*D.P.*):** During the *S.P.* period, the virus is discoverable with a set detection probability (*D.P.*) at each step. In the simulation, detected patients are immediately isolated (i.e., set aside from the system), after which they are not able to transmit the virus.
(vi) **Detection Probability during the presymptomatic phase (*DP2*):** We considered that during the *P.P.* the virus is, to some extent, discoverable if asymptomatic patients undergo a diagnostic test (e.g. a PCR test^16^). We thus introduced a second detection probability (*DP2*) for people in the *P.P*. As for the symptomatic case, detected patients are immediately isolated and, therefore, unable to transmit the virus. Additional features, such as people violating confinement or the introduction of better and faster diagnosis tests, could be explicitly factored into the model using additional parameters. However, the current model can include, to some extent, such considerations through modulation of the existing parameters.
(vii) **Simulation particulars:** The programs were encoded using Python (v.3.4.8), and random numbers were generated using the Python "rand" function. The programs were run on an 8 Xeon processor Linux server (HPC Systems, Tokyo, Japan). A 100 steps simulation with 1000 people and 2000 sites took between 90 and 190 s for completion, depending on the initial conditions.

## Results

### Probabilistic approach for simple settings (*M* = 21 and *N* = 3)

Simulation results were generated using a one-dimensional grid model. Analytical probabilities can be calculated for some simple limiting cases, and we used them to check the veracity of the simulation (Supplemental materials). We first concentrated on clarifying the relationship between personal mobility following a period of lockdown (*m*_max_ = 0) and the number of person-to-person contacts using a simplified setting (*M* = 21 and *N* = 3). We considered two general types of initial distribution (i) randomly and (ii) regularly spaced.

(i) Random initial distribution: As described in the supplementary section, when people are randomly distributed on the grid, the probability that two people are collocated at the same site at the beginning of the lockdown relaxation period (*t* = 0) is given by *P*(2, *t* = 0) and for *M* = 21 and *N* = 3 it is calculated as *P*(2,*t* = 0) = 1/7 = 0.1428, (see Supplementary section A). Relaxation of personal mobility with random displacement will not disturb the initial random nature of the distribution, and so, on average, the encounter probabilities at a later time remain unchanged (i.e., *P*(2, *t* > 0) = 0.1428, see Supplementary section A). Note that this is an equilibrium average with deviations from the mean expected for any single simulation.
(ii) Regularly-spaced initial distribution: We next considered how the encounter probability changes when the initial disposition is a regularly-spaced distribution whereby the three people are equally separated by seven distance units). For the regularly-spaced distribution, the probability of person-to-person contact at time zero is zero, i.e., *P*(2, *t* = 0) = 0. After multiple steps with restricted or non-restricted random movement, we expect that the encountering probability will reach its random value of 0.1428 (for *M* = 21, *N* = 3), and this is indeed what we observe (Fig. 1c). To investigate, in detail, the effects of mobility on the encountering probability, we calculated its value after a single time step, *P*(2,*t* = *Δt*) for mobility *m*_max_ ranging from 0 (null) to 10 (full mobility). Due to their initial separation of six distance units, only mobilities (*m*) that can span this separation distance have the capability for producing a *P*(2, *t* = *Δt*) > 0, as seen in Fig. 1c for *m*_max_ ≥ 4 (Fig. 1 and Supplementary section C).

Another intuitive way of describing this point is to note the relationship between the probability for person-to-person overlap, *P*(2, *t* = *Δt*), and the person’s density, *ρ*_L_ = *N*/*M*, and personal mobility, *m*_max_**,** is given by (Eq. 1).

1 $$P(2,t=\Delta t) \geq 0 \iff 2{m}_{\mathrm{max}} \geq1/{\rho }_{L}$$

Thus, for $2{m}_{\mathrm{max}} \geq1/{\uprho }_{L}$ the people can move freely, and it is thus expected that the contact number converges to the above asymptotic value of *P*(2,*t* = *Δt*) = 0.1428 (for *M* = 21 and *N* = 3).

### A one-dimensional grid model reproduces the basic features of the viral spread statistics (*M* = 2000 and *N* = 1000)

Next, we examined whether our model is capable of reproducing some basic features of the viral spread for a larger (and therefore slightly more realistic) system. Thus, we first assessed whether the results of our simulation are independent from the model’s size (or free from finite-size effects). To this end, we calculated the total number of infections for *M* = 20 to 10,000. The number of infections was nearly constant at around 70% of the total number of people, indicating the simulation results are indeed size-independent (Supplementary Fig. S1).

**Figure 2:**
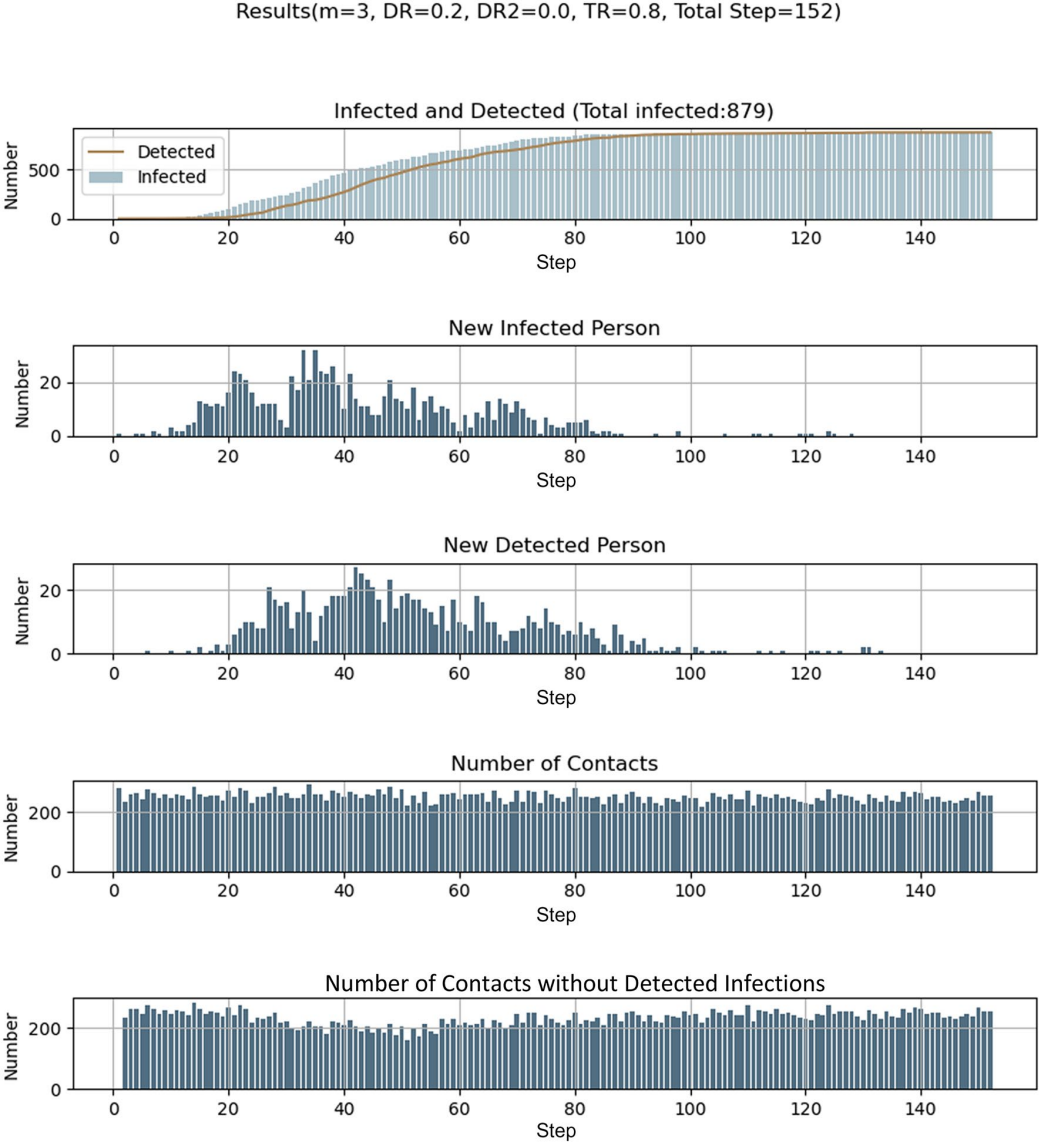
Example of time-dependent virus transmission. The parameters are as follows: *N* = 1000, *M* = 2000, *t* = 1. At the initial state, *m*_max_ was set to 100 and is reduced to *m*_max_ = 0 when the number of detected infections reaches 1% (10 people). Infected patients are infectious for 15 steps (five steps as non-detectable asymptotic persons and ten steps as a symptomatic and detectable patient). After fifteen steps, they are removed from the system: they cannot be infected nor become infectious a second time. Parameters are given in the figures.

Using a model with 1000 people and a grid of 2000 positions, we then showed that our model reproduces the basic features of an infection outbreak starting from a single presymptomatic individual seeded randomly into the system (Fig. 2). In the beginning, mobility was not restricted, and people could move within a mobility range set to *m*_max_ = 100. In our model, the lockdown was enacted by reducing the mobility from the initial *m*_max_ = 100 to 0 (or to the value given in Table 1) when the number of detected infections reached 1% of the population (10 people). This mobility restriction was relaxed after an interval of 10*Δt*, which corresponds to the time of symptomatic infection in our model. As can be noted, during the early stages, the number of infections remains low (until step 10 in Fig. 2 and S2), but after *t* = 20*Δt*, the number of new patients increases significantly (despite the average number of contacts remaining the same). An apparent second wave appears due to the fact that many undetected infectious patients remain within the system after the end of the first wave. It is noteworthy that at the end of the simulation, some people remain un-infected. This is because, in our current setting, we assumed that people are infected only once and remain infectious for only fifteen time-steps. Under this setting, the spread of infection stopped on average after *t* = 70*Δt* because no infectious patients remained in the system (Fig. 2 and S2). In epidemiological terms, this is the functional equivalent of reaching a herd immunity point in the sense that the susceptible patient number becomes dilute enough to prevent the sustaining of the infection cycle^17^.

**Table 1:** Dependency of the number of infections on mobility restriction, detection probability, and transmission probability (*N* = 1000 People, *M* = 2000 sites, 0.8 transmission probability (*T.P.*), mobility restriction applied at 1% (= 10 people)).

Detection probability		Total infection^1^			
		*m*_max_ = 0	*m*_max_ = 3	*m*_max_ = 5	*m*_max_** = **100 (initial)
0		998	997	998	997
0.2		613	879	909	946
0.4		189	727	834	906
0.6		71	601	698	847
0.8		51	521	567	810
1		49	341	426	797

^1^Total number of infected people at the end of the simulation.

### Effect of mobility restriction vs. reduction of viral transmission and the probability of detection

Next, we compared the effect of change in mobility, *m*_max_, against variation in either of the two parameters relating to detection probability (*D.P. and DP2*) and transmission probability (*T.P.*) (Tables 1–3). Similar to the simulations shown in section B, a partial lockdown involving a change in *m*_max_ was automatically instituted when the number of detected infections reached 1% of the population.

**Figure 3:**
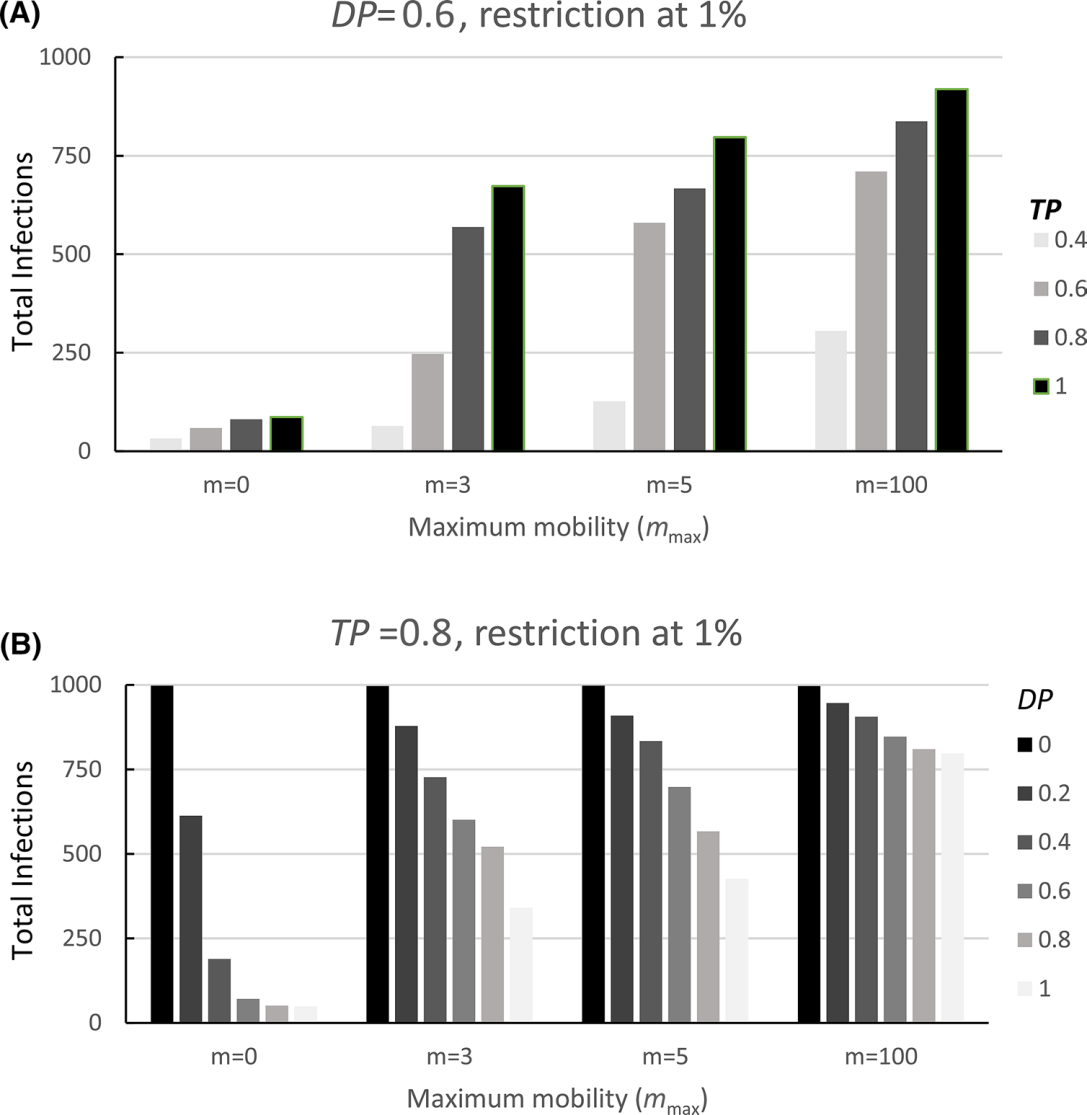
Effects of mobility restriction vs. Detection Probability (*D.P.*) and Transmission Probability (*T.P.*) on infection probability. The other parameters are as follows: *N* = 1000, *M* = 2000 and during the non-confinement period, the mobility (*m*_max_) is set to 100. The mobility was constrained when the number of detected infections reaches 1% (ten people) by setting max (*m*)) to the indicated value (*m*). People that are not detected during the 15 days of infection (5 days of asymptotic and 10 days of symptomatic infections) won’t be infectious nor be infected and are practically set apart from the system (**A**) *D.P.* was varied from 0.0 to 1.0 with *T.P.* set to 0.8. (**B**) *TP* was varied from 0.4 to 1.0 using a *DP* = 0.6. Color codes for *D.P.* and *T.P.* are given within the figures.

To investigate the importance of mobility vs. detection probability, we performed simulations in which the detection probability for symptomatic persons (*D.P.*) was varied from 0 to 1 for five mobility values. In this simulation, the detection probability for presymptomatic cases (*DP2*) was set to zero. *D.P.* is interpreted as the probability that a symptomatic patient is correctly diagnosed and subsequently isolated (meaning they cannot then infect other people). A null detection probability (*D.P.* = 0) means that symptomatic patients are neither diagnosed nor isolated. On the other hand, a 100% successful detection protocol (*D.P.* = 1) implies that all suspected symptomatic patients are systematically tested and diagnosed with perfect accuracy; and that once identified, the patients are strictly isolated. Our results suggest that the reduction of mobility is a determining factor, but that full lockdown (*m*_max_ = 0) has to be coupled with a detection probability > 0.4 to be effective (Fig. 3, Table 1). When the detection probability falls to 0.2 or less, the beneficial effects of the lockdown are significantly reduced (Fig. 3, see *m*_max_ = 0), with the total number of infections eventually increasing to values comparable to a scenario involving no lockdown but a detection probability of 0.6 to 0.8 (Fig. 3, *m*_max_ = 5). Note that the high infection levels observed for *D.P.* = 0 are due to the fact that, in our simulation, the infected patients are not detected nor isolated (*D.P.* = 0), and therefore the lockdown value of 1% of the population is never reached, and thus no lockdown implemented during the entire simulation.

**Table 2:** Dependency of the number of infections on mobility restriction and detection probability, and transmission probability (1000 people, 2000 sites, 0.6 detection probability, restriction at 1%).

Transmission probability	Total infections^1^			
	*m*_max_ = 0	*m*_max_ = 3	*m*_max_ = 5	*m*_max_ = 100 (initial)
0.4	32	64	127	305
0.6	59	247	580	710
0.8	81	569	667	837
1.0	87	673	797	919

^1^Total number of infected people at the end of the simulation.

To investigate the importance of mobility vs. transmission probability, we performed simulations in which the transmission probability (*T.P.*) was systematically varied from 0.4 to 1.0 for five different mobility values. The transmission probability (*T.P.*) is the fractional probability of infection of a non-infected person by an infected patient when the two are co-located at the same grid site during a single time period. In real life, the *T.P.* parameter can be controlled by non-pharmaceutical measures^18,19^ such as social distancing^20–22^, hand washing, and mask-wearing^23,24^. As expected, our simulations show that reducing the transmission probability will decrease the total number of infections (Table 2 and Fig. 3A, see values in lanes *m*_max_ >  = 3). Less expected was the finding that reducing *T.P.* to a value of 0.4 in combination with enforcing reduced mobility measures was as effective as enforcing a complete lockdown (Fig. 3A; *m*_max_ = 0). Inversely, a high *T.P.* (≥ 0.6) strongly increased the spread of the virus (Fig. 3A).

Finally, we examined the effect of detecting the virus at the presymptomatic phase (***P.P.)*** by setting *DP2* > 0. This approach requires a high fraction of the entire population to be tested on a regular basis, which might not be sustainable in the real world. However within the simulation, this strategy proved extremely effective because infected patients in the *P.P.* period were detected and put in quarantine very early in the infection cycle. Additionally, since the mobility reduction is decided based on the number of detected infections (rather than the actual number of infected patients), identifying these presymptomatic patients early on can assist in more closely estimating the actual number of infections in the system, thus implementing an earlier lockdown. Noteworthy, increasing *DP2* from 0 to just 0.1 can dramatically decrease the number of infections, even for a mild mobility restriction of *m*_max_ = 3 or 5 (Table 3).

**Table 3:** Dependency of the number of infections on mobility restriction and detection probability during the presymptomatic phase, and transmission probability (1000 people, 2000 sites, 0.8 transmission probability, restriction at 1%). Total number of infection for *DR2* = 0 to 0.4.

Detec Prob *PP* ^1^ (*DP2*)	Total Infections^2^ *(m*_max_ = 0)			Total Infections^2^ *(m*_max_ = 3)			Total Infections^2^ *(m*_max_ = 5)			Total Infections^2^ *(m*_max_ = 100)		
	*DR* = 0.8	*DR* = 0.6	*DR* = 0.4	*DR* = 0.8	*DR* = 0.6	*DR* = 0.4	*DR* = 0.8	*DR* = 0.6	*DR* = 0.4	*DR* = 0.8	*DR* = 0.6	*DR* = 0.4
0	59	66	110	323	562	669	452	574	792	815	863	897
0.05	38	47	57	201	280	578	273	299	632	713	739	841
0.10	23	26	32	110	128	202	111	124	381	599	653	757
0.20	13	21	28	23	33	44	45	67	76	196	223	211

^1^*DR2*: Detection probabilities at the presymptomatic phase.

^2^Total number of infected people at the end of the simulation.

## Discussion

The spread of viral infection is usually modeled using analytical models developed with different levels of complex approximations required to achieve a solution^12^. In contrast, by not seeking to develop a closed analytical form, the one-dimensional numerical approach introduced here has the merit of conceptual simplicity, and as such, we can utilize parameters more closely aligned to real-life features. The model affords us the opportunity to make both qualitative and semi-quantitative predictions for sophisticated scenarios. For example, we could readily accommodate a population with two types of people exhibiting mobility and transmission characteristics representative of, for instance, the younger and the older generations. Similarly, the model can be readily adapted to fit other bespoke confinement/de-confinement strategies for particular social situations. Such versatility is frequently unattainable with an analytical model, which tends to be generally less flexible^11,12,25^. Another advantage of using numerical simulation is that parameters, such as the reproduction ratio^25,26^, are straightforwardly determined as an exercise in simple counting. However, by taking the numerical simulation approach we do not have a simple means of using our model in regression analysis of experimental data (to calculate ‘best fit’ model parameter values), therefore our model should be taken as predictive but not analytical in nature^27^.

As with all simulations, the interpretation of the results requires caution. Seeking correspondences in the real world for our generalized descriptive variables and parameters (e.g., those relating to unit time (*Δt*), distance, physical collocation, and mobility) necessarily involves a certain degree of ambiguity. Nevertheless, despite these limitations, we believe that our model provides useful qualitative and semi-quantitative information on how virus-specific and society specific parameters influence the way viral spread occurs during periods of lockdown and re-opening. While some observations were anticipated, such as an early lockdown being more effective than a delayed one, others were less intuitive. For example, the relationship between mobility and the probability of encounter was critically dependent upon the population's initial distribution and population density.

Furthermore, our model predicts that mobility restriction must be stringently enforced and accompanied by a high detection/isolation probability to significantly reduce the total number of infections. Finally, the detection and quarantine of presymptomatic patients (which would require the regular testing of a large number of people and is therefore perhaps not a realistic strategy) would reduce the final number of infections by a factor of ten or more. This might explain the large variation in the number of infections in countries that adopted similar measures of confinement according to Hale et al.^28^ (see Supplementary Materials Section E for a discussion of “real world” data). Successful strategies for achieving a low number of infections include full confinement combined with a reasonable detection probability of symptomatic patients (*D.P.* > 0.4). A realistic strategy might be mild confinement, with a high detection probability during the symptomatic phase and a reasonable detection probability during the presymptomatic phase (for example, *m* = 5, *D.P.* = 0.8, *DP2* = 0.2 in Table 3. Such a strategy is in line with a recent report by Muller et al.^29^).

Despite the lack of an exact physical correspondence between the time and distance scales used in this model and real life we now discuss the surety of the literature parameter values reported for the infectivity parameters^30^. There is a reasonable consensus of five days for the presymptomatic phase during which the patient is infectious, but estimates for infectivity during the symptomatic period varies largely from a few days to over 20 days^31^. Our model predicts that if no presymptomatic patients are detected (*DR*2 = 0), over 90% of the infections will occur during the presymptomatic period (Supplementary Materials Fig. S-4). Furthermore, even if we lower the probability of transmission (*TP*) to 0.4 (from 0.8 in Fig. S-4a and b), most of the infections will occur before the appearance of the symptoms and the final number of infection will not decrease significantly (Supplemental materials Section F; Fig. S-4c.) This could explain, from an epidemiological viewpoint, the importance of detecting and isolating presymptomatic patients in a SARS-type outbreak, where the majority of infections appear to occur during the presymptomatic rather than the symptomatic phase^32^. This observation corroborates our calculation that even a moderate detection probability of 0.2 during the presymptomatic phase will lower the total number of infections (Table 3). To date, we are aware of some reports describing the potential for COVID-19 reinfection of previously recovered patients. Although this situation is considered uncommon, the simulation could be readily modified to include such a scenario as well as the effects of vaccination that has recently started.

Finally, we consider the trade-offs of using a one dimensional vs. a more realistic two-or three-dimensional grid. As constructed, our 1D model offers considerable advantages in terms of conceptual transparency. The simplicity of a 1D approach has allowed us to readily verify the results for small systems using exact probabilistic calculations as described in the appendix. Admittedly, the time and distance scales in our one-dimensional model do not directly relate to the temporal and spatial distances and surfaces in real life; therefore some form of scaling would be required when applying these in a quantitative manner. A possible form of this scaling might be drawn from those applied to relate results generated from simulations that utilize different dimensionalities of diffusive process^33^. However, given the coarseness of the model, it is not clear whether using a higher-dimensional representation would prove more informative than the present 1D-model and it may run the risk of losing the conceptual clarity associated with the present exposition.

## Conclusion

We presented a stochastic model where people were allowed to either move freely or in a constrained manner, and viral transmission can occur when infected and non-infected individuals overlap at the same site. Our model includes adjustable parameters characterizing viral transmission probability, detection probability, and personal mobility within a population. The correctness of our model was assessed using exact probabilistic calculations for simple limiting cases. Applied to larger systems, our stochastic simulation could reproduce basic aspects of viral spread within a community during an epidemic. Although many of the results were in line with our anticipation, our model revealed a number of interesting features. In particular, we noticed that the link between personal mobility and the risk of encounter (and thus infection) is a step function when the initial distribution is regularly-spaced, and the maximum mobility lies below the inverse spatial density of the population. The infection risks were zero below this critical juncture, whereas, at greater mobility (or higher population density), the situation rapidly approached the random case. Such a finding strongly suggests that lockdown strategies should be tailored to population densities, with the requirement for restricting mobility under lockdown therefore necessarily being different for high vs. low density populated areas. The approach described here provides a qualitative assessment of the efficacy of modifying societal parameters that should prove useful to decision-makers when considering lockdown strategies.

## Supplementary Information

Supplementary Information

## Data Availability

All data are given in the manuscript and the supplementary data. The original program can be freely accessed at http://domserv.lab.tuat.ac.jp/covid19.html.
