# Supplementary material for: Stochastic modelling of the effects of human-mobility restriction and viral infection characteristics on the spread of COVID-19
Source: Sci Rep. 2021 Mar 25;11:6856. doi: 10.1038/s41598-021-86027-2 (PMC7994631; doi:10.1038/s41598-021-86027-2)
Supplement: Supplementary file 1 — Supplementary Information [file 41598_2021_86027_MOESM1_ESM.docx]

**Supplementary Materials**

**Stochastic modelling of the effects of human-mobility restriction and viral infection characteristics on the spread of *COVID*-19**

Shiho Ando^1$^, Yuki Matsuzawa^1$^, Hiromichi Tsurui^2^, Tetsuya Mizutani^3^, Damien Hall^4^, & Yutaka Kuroda^1^


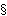


^1^ Department of Biotechnology and Life Sciences, Tokyo University of Agriculture and Technology, 2-24-16 Nakamachi, Koganei-shi, Tokyo 184-8588, Japan.

^2^Department of Immunological Diagnosis, Juntendo University School of Medicine, 2-2-1 Hongo, Bunkyo-ku, Tokyo 113-8421, Japan.

^3^ Research and Education Center for Prevention of Global Infectious Diseases of Animals, Tokyo University of Agriculture and Technology, 3-5-8 Saiwai-Cho, Fuchu-shi, Tokyo 183-8509, Japan.

^4^ Department of Life Sciences and Applied Chemistry, Nagoya Institute of Technology, Gokiso Showa. Nagoya Aichi 466-8555. Japan.

^$^ Equal contribution

Correspondence to Y.K.: Email: [ykuroda@cc.tuat.ac.jp](mailto:ykuroda@cc.tuat.ac.jp); Phone/Fax: +81-42-388-7794


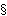


Contents: Four figures (S1 to S4), one table (S1), and one Appendix

N=10 　　　 　　N=100 　 N=1000　　 N=5000

M=20 　　　　 M=200 　 　 M=2000 M=10000

System’s size

**Figure-S1:** Size dependency. The percentage of infected patients was about 70% for systems with grid sizes ranging from 20 to 10000 positions, and a population density of 0.5. The values are averaged over 5 runs of 100 steps

**
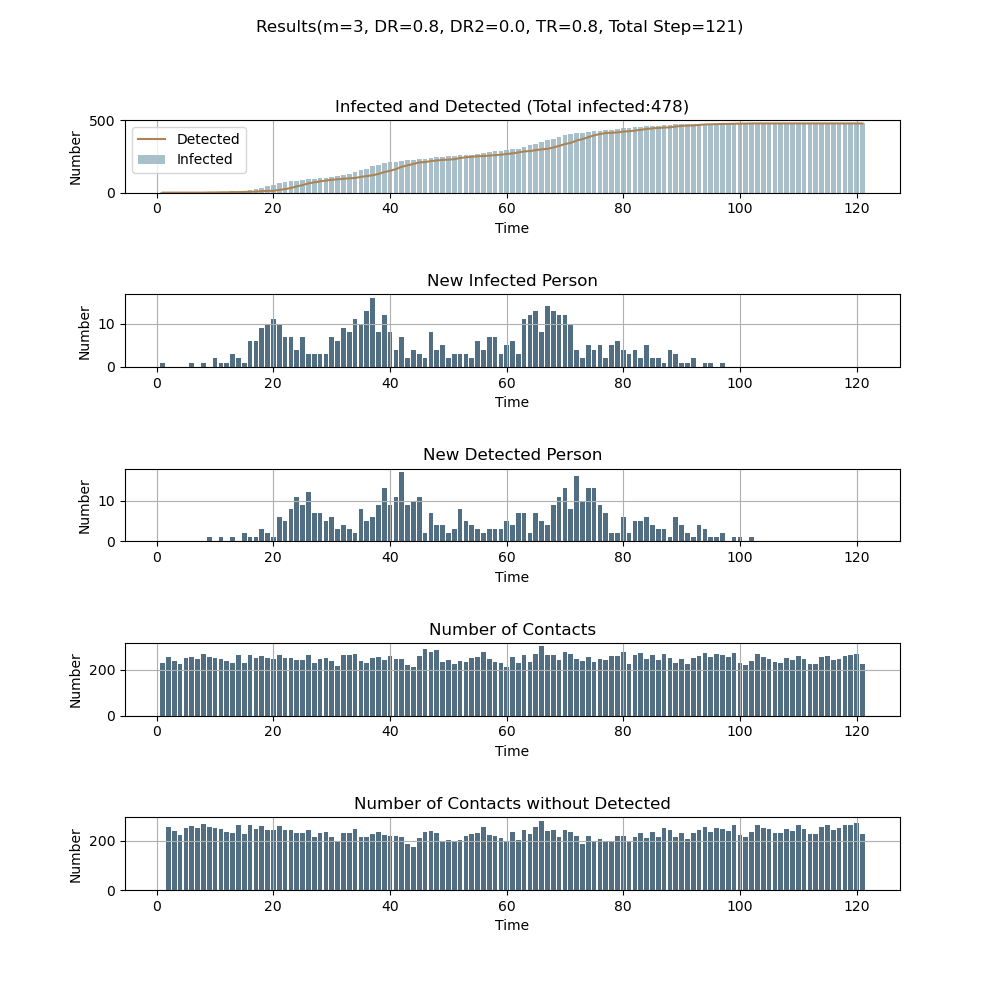
**

Infections

**Figure S-2.** Example of time-dependent virus transmission. The parameters are as follows: *N*= 1000, *M*=2000, t=1. At the initial state, *m*_max_ was set to 100 and is reduced to *m*_max_ = 1 when the number of detected infections reaches 1% (10 people). Infected patients are infectious for 15 steps (five steps as non-detectable asymptotic persons and ten steps as a symptomatic and detectable patient). After fifteen steps, they are removed from the system: they cannot be infected nor become infectious a second time. Parameters are given in the figures.

**Appendix: Limiting case results from analytical solutions and numerical simulation**

In simple cases, the probability of encounters simulated in the 1D grid model can be calculated directly or simulated using analytically derived equations. To produce such simple limiting case results we consider a grid with 21 sites (*x* positions ranging from *x* =0 to *x* = 20; *M*=21) with both ends of the grid connected in order to avoid end effects. Namely, position 20 and position 0 are adjacent (periodic boundary condition).

**(A) Encounter probabilities for randomly arranged particles**

The current work considers the case of *N* = 3 people [A, B, C] located amongst *M* = 21 sites *x* ∈ [0, 20]. Three general classifications of initial states are possible which correspond to the following distributions.

(i) Three people all located at different positions on the grid, **D_1_** = {*x_A_* ≠ *x_B_* ≠ *x_C_* }

(ii) Two people located at the same position, one at a different position, **D_2_** = {*x_A_* = *x_B_* ≠ *x_C_* }

(iii) Three people all located at the same position on the grid, **D_3_** = {*x_A_* = *x_B_* = *x_C_* }

To calculate the probability of each state we apply standard rules as follows,

$P(\mathbf{D}_{\boldsymbol{1}};M=21,N=3)=\left( \frac{M-1}{M} \right)\left( \frac{M-2}{M} \right) = 0.8617$ [A1a]

$P(\mathbf{D}_{\boldsymbol{2}};M=21,N=3)=N\left( \frac{1}{M} \right)\left( \frac{M-1}{M} \right)= 0.1361$ [A1b]

$P(\mathbf{D}_{\boldsymbol{3}};M=21,N=3)= \left( \frac{1}{M} \right)^{(N-1)}= 0.0022$ [A1c]

Allowing for multiple combinations of selection, the probability for two persons to collocate at the same position can be evaluated as the expectation value of the number of overlapping people which is given by (P(D_2_) + _2_C^3^ P(D_3_)) =1/7≈ 0.1428 . As the purpose of the present study is to examine the effect of mobility on the spread of infection following the relaxation of lockdown it is important to understand that these three distribution classifications making up the random case individually represent potentially different starting points that individually could produce different results for mild-restriction/relaxation of the mobility. However in calculating the general situation, each of these individual probabilities are used to calculate the average case.

**(B) Encounter probabilities after a single step for regularly arranged particles with restricted mobility**

Here we formally consider the distribution of persons in terms of discrete probability density function P_A_(x), P_B_(x), P_C_(x) for persons A, B and C. For an arbitrary grid position x, the density function for the random case will be described by a flat distribution corresponding to Eqn. A2 where Q ∈ [A,B,C].

$P_{Q}(x, t=0)= \left( \frac{1}{M} \right)$ [A2]

In the above example, where the three people were randomly arranged, the effective probability of encounter was seen to be independent of the person’s mobility. Here we consider an example where the people are initially placed at a regular distance, z, from each other. In this case, rather than being a flat distribution, the probability density function is periodic in nature (Eqn. A3).

$P_{Q}(x, t=0)=\left\{ \begin{matrix} \left( \frac{1}{N} \right) for x = i.z \\ 0 for x \neq i.z \end{matrix} \right. where (i \in[0,1,2])$ [A3]

For the case of *N* = 3, *M* = 21 and *z* = 7 we have P_Q_(x=0,7,14) = 1/3 with P_Q_(x) at all other positions = 0. Again we consider the case of a person moving from their original position x, during the time interval, Δt, with a step size j taken without bias from the set [−*m*_max_, *m* _max_]. After movement associated with a single time period the distribution will flatten but retain its periodic nature for maximum step sizes characterized by *m*_max_ < z.

$P_{Q}(x, t=t)=\left\{ \begin{matrix} \left( \frac{1}{N} \right)\left( \frac{1}{2m+1} \right) for x [\left( i.z-m \right),\left( i.z+m \right) ] \\ 0 for x \ni[\left( i.z-m \right),\left( i.z+m \right) ] \end{matrix} where m\max< z \right.$[A4]

For an initially regular periodic arrangement, Eqn. A5a describes the probability of overlap between persons A and B at one considered shared region of overlap. With reference to the initial assignment of persons A and B Eqns. A5b and A5c describe the probability of encounter between B and C and C and A at the other regions of overlap. Eqn. A5d describes the total probability of any form of overlap between all regions on the grid.

$P_{A and B}(t=t)=\left\{ \begin{matrix} 0 for m (z-1)/2 \\ N\left( \frac{1}{N} \right)\left( \frac{1}{N-1} \right)\frac{\left( 2m+1-z \right)}{\left( 2m+1 \right)^{2}} \end{matrix}for m > (z-1)/2 \right.$ [A5a]

$P_{B and C}(t=t)=\left\{ \begin{matrix} 0 for m (z-1)/2 \\ N\left( \frac{1}{N-1} \right)\left( \frac{1}{N-2} \right)\frac{\left( 2m+1-z \right)}{\left( 2m+1 \right)^{2}} \end{matrix}for m > (z-1)/2 \right.$ [A5b]

$P_{C and A}(t=t)=\left\{ \begin{matrix} 0 for m (z-1)/2 \\ N\left( \frac{1}{N-2} \right)\left( \frac{1}{N} \right)\frac{\left( 2m+1-z \right)}{\left( 2m+1 \right)^{2}} \end{matrix}for m > (z-1)/2 \right.$ [A5c]

$P_{A and B}+P_{A and C}{+ P}_{B and C}=\left\{ \begin{matrix} 0 for m (z-1)/2 \\ N\frac{\left( 2m+1-z \right)}{\left( 2m+1 \right)^{2}} \end{matrix}for m > (z-1)/2 \right.$ [A5d]

The situation described above was also simulated numerically as described in Section D with the initial conditions adjusted to allow for a well separated arrangement of people at zero time. As can be seen the behavior predicted by Eqn. A5 is identical to that produced by the simulation (**Fig. S3**).

**(C) Encounter probability for randomly arranged particles with restricted mobility**

As shown in section A, for a completely random distribution the probabilities of distributions **D_1_**, **D_2_** and **D_3_** occurring are given by Eqn. A1a-c. For this population (defined by a grid size of *M* = 21 that contains *N* = 3 people), in the limit of small personal mobility, such that *m_max_* = 0, the random distribution properties developed in section A would remain unchanged. Likewise for the same population in the limit of large personal mobility sufficient to completely traverse the grid (such that *m*_max_ ≥ 10) we would also expect the distributions produced after movement to also be random. In the intervening range we strongly suspect that the probabilities associated with cases of limited personal mobility (*m*_max_ ≤ 10) are also likely to be random^[[1]](#footnote-1),^^[[2]](#footnote-2)^

**(D) Numerical calculation of the distribution produced by overlaying limited random mobility** **(case B and C)**:

As shown, for a completely random distribution the probabilities of distributions **D_1_**, **D_2_** and **D_3_** are given by Eqn. A1a-c. For the same population (defined by a grid size of *M* = 21 that contains *N* = 3 people) in the limit of large personal mobility, such that ***m***_max_ ≥ 10, we would expect the distributions produced after movement to also be random (see case B). To further confirm the nature of the distribution produced by overlaying limited random mobility to an initially random distribution we used an independent form of numerical simulation to the model described in the paper. To each of one-thousand initial random distributions, ten-thousand cycles of random addition were carried for each of the three particles with elements chosen without bias from a particular mobility set ***m***, the maximum of which ranged from 0 to 10 with the results shown in the Figure below. To carry out these simulations a 21 segment circular grid was assigned. For each particular random case considered, the initial positions of the three people on the circular grid was assigned by randomly choosing a value of m from 0 to 20 using an appropriately initialized default random number generator supplied in MATLAB R16b (Mathworks, Massachusetts). Overlap was judged using a logical assignment criteria based on the position of each person on the grid. After establishing an initial random position a mobility value, j, was selected from the set **m** and the person’s position adjusted via addition. After each cycle of random addition the position of each particle was determined by calculating the modulus in relation to the grid size and the overlap determined. As can be seen for each limited mobility the probabilities produced for each of the three types of distributions (**D_1_, D_2_** and **D_3_**) were identical (within error) to the random case (Fig. S-3).


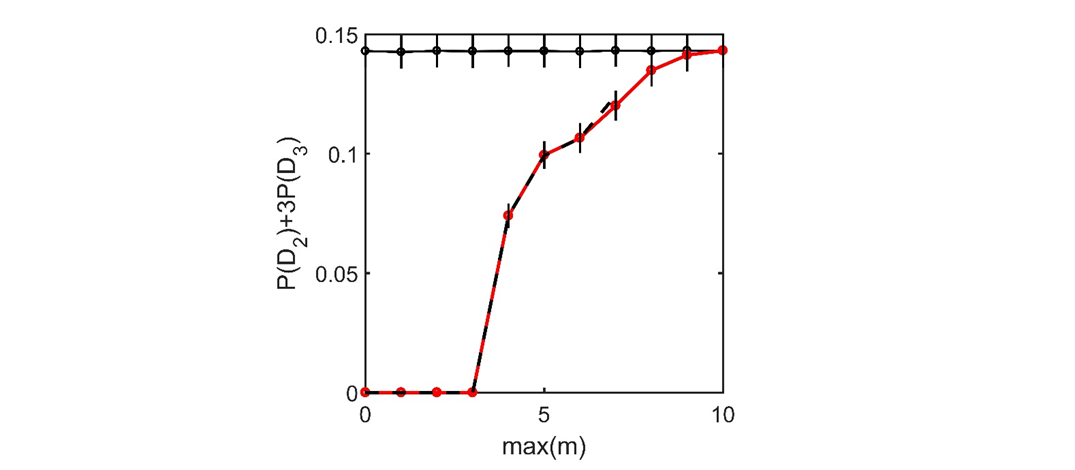


**Figure S-3**: Analytical and numerical results reflecting the probability of personal encounter as a function of personal mobility for a population that was initially arranged in either a completely random fashion or a well-dispersed regular arrangement. The population consisted of N = 3 people arranged on a grid of size of M =21. Each person’s mobility was assigned by flat sampling from a mobility set **m** = [-m,m]. The solid black line connecting black circles displays the results simulated in section B for the case of increasing extents of personal mobility available to an initially randomly arranged population. These simulated results agree with the exact analytical result of 0.1428 calculated from Eqn. A1. The dashed black line ranging from *m*_max_ = 0 to 7 indicates behavior predicted by Eqn. A5 for the probability of encounter for the regularly distributed arrangement described in section C, in which each person was initially separated by seven grid positions. The analytical result is in agreement with the simulated results for the well dispersed case shown by the solid red lines. Error bars for all the simulated results represent two standard deviations.

(**E) Table S-1 Stringency index, confirmed cases for the March-May 2020 period of representative countries.**

|  | **Stringency**  **(average for March1-21)** | **Stringency ^1^**  **(average for March 1 ~ May 31)** | **Confirmed^2^ cases per 1 million (on July/1 2020) (normalized by setting 0 on March 1)** | **Simulation Tot**  **per 1 million ^3^**  **(average *m*_max_ )** | **Simulation Tot**  **per 1 million ^4^**  **(average *m*_max_)** |
| --- | --- | --- | --- | --- | --- |
| **Brazil** | **30%** | **66%** | **6815.75** | **823200 (70)** | **612600 (34)** |
| **Germany** | **35%** | **63%** | **2336.52** | **817800 (65)** | **666800 (37)** |
| **France** | **55%** | **78%** | **2985.21** | **786250 (45)** | **420200 (22)** |
| **United Kingdom** | **17%** | **63%** | **4178.71** | **829600 (83)** | **630400 (37)** |
| **Japan** | **41%** | **43%** | **146.90** | **805000 (59)** | **803200 (57)** |
| **Sweden** | **18%** | **52%** | **6791.98** | **820750 (82)** | **784200 (48)** |
| **United States** | **33%** | **63%** | **8097.46** | **795250 (67)** | **561600 (26)** |

^1^ Adapted from Thomas Hale, Sam Webster, Anna Petherick, Toby Phillips, and Beatriz Kira. (2020). Oxford COVID-19 Government Response Tracker. Blavatnik School of Government

^2^ The total number of infections were retrieved from Dong E, Du H, Gardner L. An interactive web-based dashboard to track COVID-19 in real-time. THE LANCET Infectious Diseases. 2020 Feb 19

^3^ Infection numbers calculated by setting the maximal mobility to 0-100 as converted from the stringency index during the entire simulation. The stringency was converted to *m*_max_ as follows: *m*_max_=0 for Stringency =100; and *m*_max_ =100 for stringency= 0) converted from the stringency index for March 1-21. *DP*=60%, *TP=*80% and *TPP*=0% was used. The stringency quantifies the severity of the lockdown, mainly in terms of mobility restriction (but not solely).

^4^ Infection number calculated with the same parameter as ^3^ but with stringency index averaged over the March1-May 31 period.

Table S-1: Number of infections as reported by the CDC and the stringency factor of the non-pharmaceutical measures imposed during the March to May period as defined by T. Hale et al ^1^. For many countries, the stringency indices correlate with the number of reported infections (per capita), but there are counterexamples. Counterexamples are countries that enforced strict confinement but did experience many infections or those that enforced a soft lockdown but kept a low number of infections. Some of the discrepancies might be due to the way statistics were gathered. Still, our model suggests that one needs factors other than a restriction on *m*_max_ to rationalize the considerable variation, such as presymptomatic detection or a lowering of the transmission rate, as discussed in the main text.

(F) Simulation with “real-world” epidemiological parameters taken from reference [29] in the main text.

Figure S-4a: Dependency of the total infection number on the infectious period. *DP* = 0%, *TP*=80%, *DP2*=0% 1000 people on a grid with 2000 sites was used. The maximal mobility (*m*_max_)was set to 100. No mobility restriction was applied was applied during the whole simulation.

Figure S-4b: Dependency of the total infection number on the infectious period. *DP* = 60%, *TP*=80%, *DP2*=0% 1000 people on a grid with 2000 sites was used. The maximal mobility was set to 100. No mobility restriction was applied during the whole simulation.

Figure S-4c: Dependency of the total infection number on the infectious period. *DP* = 0%, *TP*=40%, *DP2*=0% 1000 people on a grid with 2000 sites was used. The maximal mobility was set to 100. No mobility restriction was applied during the whole simulation.

Figure S-4d: Dependency of the total infection number on the infectious period. *DP* = 60%, *TP*=40%, *DP2*=0% 1000 people on a grid with 2000 sites was used. The maximal mobility was set to 100. No mobility restriction was applied during the whole simulation.

1. Indeed as the boundaries are periodic and the mobility is equally weighted in both the negative and positive directions it is tempting to consider that the assumption of randomness could be asserted as

   axiomatic. [↑](#footnote-ref-1)
2. Let　us define the displacement as *d* and the expectation of a particle to be at a given site after a single time step *Δt* as Move (*d*). Move(*d*) is a probability mass function, which depends only on *d*, and summation over all of the sites (0~*M*) will yield unity. Thus, for particles randomly distributed on a grid with probability (*p*), the probability of a particle to be found at a given site after time step *Δt* with displacement *d with equal probability (*1/(2**m*_max_+1*) for any d)* smaller than the maximal mobility (*d*<*m*_max_) is independent on *d* and *m* and is equal to the population density *p.* The encountering probability of two particles can then be computed using equations derived above in section A. [↑](#footnote-ref-2)
